# Sustainability and Impact of an Antimicrobial Stewardship Program on Broad-Spectrum Antibiotic Consumption in South Korea: A 14-Month Extended Follow-Up Study

**DOI:** 10.3390/antibiotics15060525

**Published:** 2026-05-22

**Authors:** Tae-Hoon No, Kyeong Min Jo

**Affiliations:** Department of Infectious Diseases, Inje University Haeundae Paik Hospital, Busan 48108, Republic of Korea; tae-hoon.no@paik.ac.kr

**Keywords:** antimicrobial stewardship program, carbapenems, piperacillin/tazobactam, days of therapy

## Abstract

Background: Antimicrobial stewardship programs (ASPs) are critical for promoting rational antibiotic use. While early implementation outcomes have been reported, extended follow-up sustainability and the impact on high-priority broad-spectrum antibiotics in South Korean secondary/tertiary hospitals require further validation. This study aimed to evaluate the extended outcomes and sustainability of an ASP over a 14-month period. Methods: This retrospective, single-center study analyzed ASP activities from January 2025 to February 2026 at a tertiary hospital in South Korea. Interventions included prospective audit and feedback (PAF) for restricted antibiotics and recommendations for prolonged prescriptions (≥14 days). Primary outcomes were the monthly rejection rate of restricted antibiotics and the acceptance rate of ASP interventions. Secondary outcomes included the days of therapy (DOT) per 1000 patient–days for meropenem and piperacillin/tazobactam (Pip/Taz). Results: During the 14-month period, the ASP intervention acceptance rate increased significantly from a mean of 72.0% in the implementation phase (January–April 2025) to 81.2% in the stabilization phase (May 2025–February 2026) (*p* = 0.035). The DOT for Pip/Taz decreased significantly from 169.4 to 151.8 per 1000 patient–days (*p* = 0.002), with a significant negative correlation identified between the intervention acceptance rate and Pip/Taz consumption (r = −0.625, *p* = 0.017). Although overall meropenem DOT showed seasonal fluctuations without reaching statistical significance across phases, a year-over-year comparison revealed a 7.5% reduction in meropenem DOT (January–February 2025: 54.8 vs. January–February 2026: 50.7 per 1000 patient–days). The rejection rate for restricted antibiotics declined from 3.8% to 2.6%, suggesting that clinicians increasingly self-regulated inappropriate prescribing attempts. Conclusions: The ASP demonstrated extended follow-up sustainability with a significant reduction in the consumption of key broad-spectrum antibiotics. A progressive increase in clinician acceptance of ASP interventions from 72.0% to 81.2%, combined with a concurrent decline in the restricted antibiotic rejection rate, reflected a measurable shift in institutional prescribing culture and confirmed the successful transition to a stabilized program. These findings support the necessity of sustained multidisciplinary ASPs, even in resource-limited settings, to combat antimicrobial resistance effectively.

## 1. Introduction

Antimicrobial resistance (AMR) is one of the most pressing global public health threats of the 21st century, accelerated by the overuse and misuse of antibiotics across healthcare, agriculture, and community settings. A landmark systematic analysis estimated that bacterial AMR was directly responsible for approximately 1.27 million deaths worldwide in 2019, surpassing the mortality burden of HIV/AIDS and malaria [1]. To address this crisis, healthcare institutions worldwide are strongly encouraged to implement Antimicrobial Stewardship Programs (ASPs) to promote the rational use of antibiotics, improve clinical outcomes, and reduce resistance rates. Core strategies include formulary restriction, prior authorization for high-priority antibiotics, prospective audit and feedback (PAF), and clinician education [2]. Despite the growing global adoption of ASPs, long-term outcome data from Asian hospitals remain scarce despite the region’s substantial antimicrobial resistance burden. A recent systematic review of hospital ASPs found that the majority of available evidence originates from Europe and North America, with limited representation from Asian and other developing regions—underscoring the need for localized, real-world evaluations from non-Western healthcare settings [3]. The Korean healthcare context presents unique implementation challenges distinct from Western systems, including high inpatient volumes, a recognized shortage of infectious disease specialists, and fee-for-service reimbursement structures that may incentivize broader antibiotic use [4]. These contextual factors make program-level evidence from Korean institutions particularly valuable for informing regionally tailored stewardship strategies.

Among the antibiotics most frequently targeted by stewardship interventions, carbapenems and piperacillin/tazobactam (Pip/Taz) are of particular concern. Both are classified as “Watch” category antibiotics by the WHO AWaRe framework, indicating high resistance potential and a critical need for stewardship oversight [5,6].

In South Korea, ASP implementation has accelerated in recent years, supported by national policy initiatives and funding from the Korea Disease Control and Prevention Agency (KDCA) [7]. In 2022, a multidisciplinary expert consensus established the first Korea-specific core elements for hospital ASPs, underscoring the growing institutional recognition of stewardship as a patient safety priority [8]. However, real-world data on sustained program performance and the sustained impact of ASP interventions on specialized antibiotic consumption remain scarce. Moreover, the Korean healthcare environment faces a well-documented shortage of infectious disease specialists and dedicated ASP pharmacists, posing a fundamental challenge to maintaining the sustainability and qualitative consistency of stewardship activities [9,10].

We previously reported the early outcomes of an ASP during the first four months of implementation at a tertiary hospital in South Korea [11]. That study confirmed the feasibility of the program, demonstrating stabilization of the restricted antibiotic rejection rate and a progressive increase in clinician acceptance of interventions. However, the short observation period precluded adequate assessment of seasonal variation and lacked sufficient statistical power to demonstrate a significant reduction in the Days of Therapy (DOT) for key broad-spectrum antibiotics, including carbapenems and Pip/Taz.

Therefore, the present study extends the observation period to 14 months (January 2025 to February 2026) to address these limitations. The objectives are twofold: first, to evaluate the longitudinal evolution of clinician acceptance rates and restricted antibiotic management indicators as the ASP transitions from an implementation phase to a stabilization phase; and second, to determine whether program stabilization translates into a measurable reduction in the consumption of carbapenems and Pip/Taz. Through this analysis, we aim to demonstrate that even in a resource-limited environment with a shortage of infectious disease specialists, a well-structured EMR-based ASP can fulfill a sustainable and effective ‘gatekeeping’ role—whereby clinicians increasingly self-regulate inappropriate prescribing attempts as familiarity with prior authorization criteria grows.

## 2. Results

### 2.1. Restricted Antibiotic Prescription and Rejection Rates

During the 14-month study period (January 2025–February 2026), the ASP team continuously monitored the appropriateness of restricted antibiotic prescriptions across all clinical departments. During the implementation phase (January–April 2025), the average number of restricted antibiotic prescription requests was 546.0 per month. In the stabilization phase (May 2025–February 2026), the volume of requests substantially increased to a monthly average of 749.4—a 37.3% rise compared to the implementation phase. Despite this increase, the average monthly rejection rate declined from 3.8% to 2.6% (Table 1), suggesting the emergence of a voluntary “gatekeeping” effect wherein clinicians became progressively more familiar with prior authorization criteria, thereby self-regulating inappropriate prescription attempts. Monthly rejection rates by individual carbapenem agent are detailed in Table 2.

### 2.2. ASP Intervention Acceptance Rate

**Figure 1:**
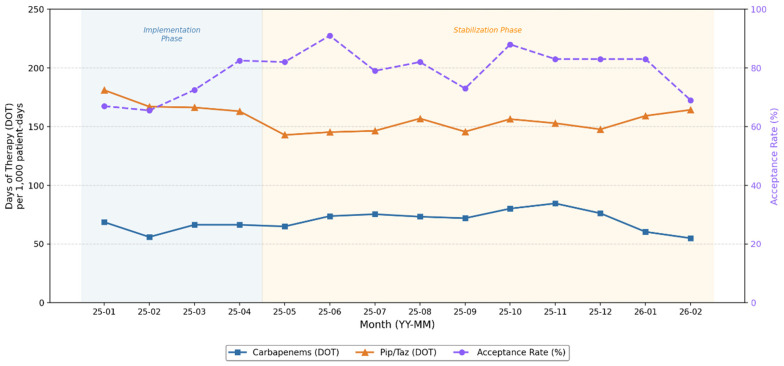
Trends in Antibiotic DOT and ASP Intervention Acceptance Rate.

Following the prospective audit and feedback (PAF) provided by the ASP team for both restricted antibiotic prescriptions and prolonged antibiotic use (≥14 days), clinician acceptance of interventions increased significantly over time (Figure 1). In January 2025, the first month of program implementation, the acceptance rate was 67.0%. Through continuous monitoring and multidisciplinary communication, the rate improved gradually, reaching a peak of 91.0% in June 2025. Comparing the two phases, the mean acceptance rate increased from 72.0% during the implementation phase to 81.2% during the stabilization phase, representing a statistically significant improvement (*p* = 0.035).

### 2.3. Antibiotic Consumption: Piperacillin/Tazobactam

**Figure 2:**
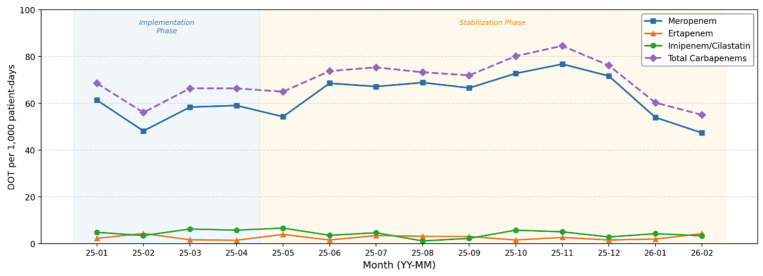
Monthly DOT by Individual Carbapenem Agent (per 1000 patient–days, January 2025–February 2026).

The impact of ASP interventions on antibiotic consumption was assessed using Days of Therapy (DOT) per 1000 patient–days for piperacillin/tazobactam (Pip/Taz) and meropenem (Figure 1 and Figure 2). Pip/Taz, which was the primary target of stewardship optimization, demonstrated a substantial and statistically significant reduction in consumption. The mean DOT for Pip/Taz decreased from 169.4 per 1000 patient–days in the implementation phase to 151.8 in the stabilization phase (*p* = 0.002). Pearson correlation analysis further confirmed a significant negative correlation between the clinician acceptance rate and Pip/Taz consumption (r = −0.625, *p* = 0.017), indicating that increased adherence to ASP recommendations directly contributed to reduced use of this antibiotic.

### 2.4. Antibiotic Consumption: Meropenem

For meropenem, which was selected as the representative carbapenem agent given its predominant use at this institution—accounting for a mean of 81.0% of total carbapenem prescription requests throughout the study period, followed by imipenem/cilastatin (5.5%) and ertapenem (3.6%) (Table 2)—the overall phase comparison did not reach statistical significance (*p* = 0.140), largely attributable to seasonal increases in consumption observed between June and November 2025, peaking in November at 76.8 per 1000 patient–days (Figure 2). This pattern likely reflects an increased influx of severely ill patients during the latter half of the year, consistent with the trends observed at our institution in the prior study period [11]. Monthly DOT trends by individual agent demonstrated that meropenem was the primary driver of overall carbapenem consumption fluctuations, whereas ertapenem and imipenem/cilastatin showed comparatively stable and low-level utilization throughout the study period (Figure 2). Nevertheless, a year-over-year (YoY) comparison—January–February 2025 (mean DOT 54.8 per 1000 patient–days) versus January–February 2026 (mean DOT 50.7 per 1000 patient–days)—revealed a 7.5% reduction, suggesting that extended follow-up stewardship efforts are meaningfully curtailing meropenem overuse.

### 2.5. Aggregate Microbiological Surveillance Indicators

To evaluate whether reductions in targeted antibiotic consumption were accompanied by compensatory shifts in healthcare-associated infection metrics, aggregate-level microbiological surveillance data were examined throughout the study period (Supplementary Figure S1).

Monthly *Clostridioides difficile* infection (CDI) incidence remained largely stable across the two phases (implementation phase mean: 1.10 per 1000 patient–days; stabilization phase mean: 1.03 per 1000 patient–days). A year-over-year comparison between January 2025 and January 2026 demonstrated a 40.5% reduction in CDI incidence (1.48 → 0.88 per 1000 patient–days) (Supplementary Figure S1A).

Among multidrug-resistant organisms (MDROs), multidrug-resistant *Acinetobacter baumannii* (MRAB) showed the most pronounced year-over-year reduction (54.4%; 0.956 → 0.436 per 1000 patient–days), followed by multidrug-resistant *Pseudomonas aeruginosa* (MRPA; −37.6%; 0.873 → 0.545 per 1000 patient–days). MRSA and VRE incidence remained essentially stable year-over-year (+1.1% and −4.7%, respectively) (Supplementary Figure S1B). CRE incidence was transiently elevated during the stabilization phase (mean 1.68 per 1000 patient–days) compared to the implementation phase (mean 1.37 per 1000 patient–days), coinciding with the period of increased patient acuity and carbapenem use observed between May and November 2025; however, on a year-over-year basis, CRE incidence showed a 12.3% reduction (1.22 → 1.07 per 1000 patient–days).

## 3. Discussion

This study evaluated the 14-month sustained outcomes of an ASP implemented at a tertiary hospital in South Korea, extending the observation period of our previously reported 4-month feasibility study [11]. While the prior study confirmed the initial feasibility of the program and the stabilization of rejection rates for restricted antibiotics, its short observation window precluded demonstration of sustained changes in prescribing behavior and statistically significant reductions in antibiotic consumption. The present study addresses these limitations directly, confirming that the ASP successfully transitioned from an initial implementation phase into a well-established stabilization phase—and, most importantly, that this transition was accompanied by meaningful and quantifiable reductions in the consumption of key broad-spectrum antibiotics.

The most clinically significant findings of this study are the statistically significant increase in clinician acceptance of ASP interventions (72.0% → 81.2%, *p* = 0.035) and the concurrent reduction in Pip/Taz DOT (169.4 → 151.8 per 1000 patient–days, *p* = 0.002). Crucially, the significant negative correlation identified between the acceptance rate and Pip/Taz consumption (r = −0.625, *p* = 0.017) provides direct evidence that the behavioral change driven by PAF—rather than formulary restriction alone—was the principal mechanism of consumption reduction. This finding aligns with growing evidence that PAF programs exert their stewardship effect primarily through clinician education and relationship-building, rather than through restrictive gatekeeping [12,13]. In a Canadian multicenter study, 82% of PAF recommendations for restricted antibiotics were accepted over a 3-year period, and physician-led audits were independently associated with higher acceptance—underscoring the value of multidisciplinary team composition in sustaining program effectiveness [14]. Similarly, a long-term 8-year Japanese study demonstrated that pharmacist-led PAF was associated with consistently high acceptance rates of approximately 90% and a sustained reduction in prolonged intravenous antimicrobial use [15]. Our observed trajectory from 67.0% to a stabilized mean of 81.2%—peaking at 91.0% in June 2025—reflects a comparable maturation curve in the context of a newly established program.

The downward stabilization of the rejection rate, despite a 37.3% increase in monthly prescription volume, is a particularly noteworthy indicator of program sustainability. This pattern suggests that the EMR-based prior authorization process functioned not merely as a gatekeeping mechanism, but as a sustained educational intervention that shaped prescribing culture over time. Repeated engagement with the authorization workflow may have progressively reinforced institutional criteria for appropriate antibiotic use among prescribers, potentially fostering a degree of self-regulation at the point of prescription—though this mechanism remains hypothetical and was not directly measured in the present study. This pattern—whereby systemic reinforcement reduces inappropriate prescribing attempts without requiring direct rejection by the ASP team—is consistent with the behavioral change framework emphasized in contemporary stewardship guidelines, which highlight that the long-term goal of ASPs is to embed appropriate prescribing into routine clinical culture [2,5]. Notably, this educational gatekeeping effect was achieved with a team of only four to five members throughout the study period, which has direct implications for resource planning in similar settings.

For meropenem, the primary carbapenem target, the phase-to-phase comparison did not reach statistical significance (*p* = 0.140), largely attributable to seasonal increases in DOT observed between June and November 2025, peaking in November at 76.8 per 1000 patient–days. This seasonal increase is likely attributable to an increased influx of severely ill patients during the latter half of the year, and is supported by concurrent institutional data: monthly meropenem prescription requests increased by 34.5% from a Q1 mean of 196 to a Q3 mean of 264 (Table 2), monthly patient admissions rose by 10.4% over the same period, and CRE incidence—a recognized surrogate marker of patient acuity and broad-spectrum antibiotic pressure—increased concurrently from a Q1 mean of 1.37 to a Q3 mean of 1.71 per 1000 patient–days. These parallel trends suggest that the seasonal meropenem increase was driven primarily by greater clinical demand rather than by deterioration in prescribing appropriateness, a pattern consistent with the trends observed at our institution in the prior study period [11].

Critically, carbapenem use is also inherently less responsive to stewardship reduction efforts, given that it is primarily driven by the clinical severity of incoming patients rather than by prescriber preference alone—a dynamic distinct from that of Pip/Taz, which is more frequently subject to discretionary prescribing [16]. Nevertheless, the year-over-year comparison between January–February 2025 and January–February 2026 demonstrated a 7.5% reduction in meropenem DOT (54.8 → 50.7 per 1000 patient–days), suggesting that sustained stewardship efforts are progressively curtailing carbapenem overuse, even if the effect is not yet detectable across the full 14-month period. This finding is consistent with accumulating evidence that carbapenem reductions through ASP interventions tend to emerge gradually over time rather than immediately following program implementation. A Korean multicenter interrupted time-series analysis at Asan Medical Center demonstrated that PAF-based carbapenem stewardship produced a significant stepwise reduction in consumption over a 5-year period, with the effect becoming statistically robust only after sustained program operation [17]. Similarly, a Swiss multicenter carbapenem stewardship initiative reported a 14.6% year-over-year reduction in total carbapenem DDD—with meropenem accounting for the largest share of the decrease (−20.9%)—within the first year of structured ID-led review [18]. In a Spanish teaching hospital, carbapenem prescription appropriateness improved from 49.7% to 80.9% over five years of PAF, accompanied by reductions in hospital-acquired MDR bloodstream infections [19]. Taken together, these findings suggest that a 7.5% meropenem reduction within the first 14 months of program operation, despite seasonal confounders, is consistent with the trajectory of maturation observed in comparable stewardship programs internationally.

To assess whether the observed reductions in targeted antibiotic consumption were accompanied by a “squeezing the balloon” effect, aggregate-level microbiological surveillance data were examined. Overall, these findings did not suggest compensatory displacement of antibiotic use or worsening of healthcare-associated infection metrics. The year-over-year reductions in MRAB and MRPA incidence are particularly notable, as both organisms are strongly associated with carbapenem and Pip/Taz selective pressure, providing indirect support for the ecological benefit of the observed consumption reductions. CDI incidence also showed a year-over-year decline, consistent with the hypothesis that reductions in broad-spectrum antibiotic use contribute to decreased *C. difficile* ecological pressure [20,21].

The transient CRE elevation observed during the stabilization phase is unlikely to represent a direct consequence of ASP interventions. CRE acquisition is multifactorial and strongly influenced by patient case-mix, contact precaution compliance, and environmental factors—all of which were simultaneously monitored and actively managed by the infection control team throughout the study period [22,23,24,25]. Similarly, the year-over-year increase in MRPA warrants continued surveillance but should be interpreted in the context of concurrent patient acuity trends rather than as a stewardship-attributable finding.

These microbiological findings should be interpreted with caution, as confounding by changes in patient case-mix, infection control activities, and surveillance intensity cannot be excluded, and a causal relationship between ASP interventions and MDRO trends cannot be established from this observational analysis.

The present study also carries important implications for ASP implementation in the Korean healthcare context, which faces well-documented structural challenges. South Korea has a recognized shortage of infectious disease specialists relative to its hospital volume, with estimates suggesting that available ID physician capacity falls substantially below the personnel requirements for full ASP coverage of hospitalized patients [9,10]. Despite operating with a team of only two infectious disease specialists, one pharmacist, and one ASP nurse throughout most of the study period, our program achieved acceptance rates and consumption reductions comparable to those reported in larger, more resource-intensive programs internationally. This finding is consistent with a growing body of international evidence supporting the viability of small-team, technology-assisted ASPs across diverse healthcare settings. In a Peruvian oncology center operating under resource-limited conditions, a similarly structured program achieved an intervention acceptance rate of 65–95% over seven years, accompanied by an 84% reduction in meropenem use—demonstrating that sustained stewardship impact is attainable even without extensive dedicated infrastructure [26]. In the Asia–Pacific region, where infectious disease specialist shortages and heterogeneous EMR adoption present challenges analogous to those in Korea, survey data indicate that EMR-based decision support and real-time alert systems are increasingly recognized as essential compensatory mechanisms for under-resourced ASP teams [27]. A long-term Japanese study at a cancer center further demonstrated that a small multidisciplinary ASP team—initially comprising a single ID physician and a pharmacist—was able to sustain meaningful reductions in broad-spectrum antibiotic consumption over 46 months through a structured notification and audit system [28]. Taken together, these international examples suggest that the EMR-based, small-team model demonstrated in this study is not unique to the Korean context, but rather represents a broadly replicable approach for healthcare systems seeking to establish sustainable stewardship programs in the absence of full-time dedicated personnel. The efficiency gains from EMR-mediated prior authorization—which enabled real-time, asynchronous communication between the ASP team and prescribers across all departments—were likely central to the program’s sustainability with a limited workforce, a dynamic that mirrors the informatics-supported stewardship framework increasingly advocated by the CDC and international stewardship guidelines [29].

This study has several limitations that should be acknowledged. First, as a retrospective, single-center study conducted at a tertiary university hospital, the generalizability of these findings to other healthcare settings may be limited. The results reflect the specific institutional context, patient population, and organizational culture of a single institution, and may not be directly comparable to smaller Korean community or secondary hospitals with fewer infectious disease specialists, more limited EMR infrastructure, and different patient case-mix. In such settings, the acceptance rates and consumption reductions observed here may be more difficult to achieve and sustain. Multicenter validation across hospitals of varying sizes and resource levels is needed to confirm the broader applicability of the EMR-based, small-team stewardship model demonstrated in this study. Second, the study assessed process metrics—antibiotic consumption and intervention acceptance rates—but did not directly evaluate ultimate clinical outcomes such as in-hospital mortality or length of stay. Although aggregate-level microbiological surveillance data were examined, patient-level MDRO acquisition rates and clinical outcomes were not assessed, and causal inference between ASP interventions and these microbiological trends cannot be established. While reductions in broad-spectrum antibiotic use are well established as proximate determinants of resistance and clinical outcomes [13,30], demonstrating these downstream effects requires longer observation periods and patient-level data collection beyond the scope of the current analysis. Third, the study design does not allow for statistical control of temporal confounders—such as changes in patient case-mix severity, local infection epidemiology, or concurrent infection control interventions—that may have influenced antibiotic consumption trends independently of the ASP. Future studies incorporating interrupted time-series analysis or matched control designs would provide stronger causal inference. Fourth, the study did not assess whether reductions in antibiotic DOT were partly attributable to adverse drug reactions or treatment-related complications, which could theoretically contribute to earlier discontinuation independent of ASP interventions. This represents a further limitation of the aggregate-level DOT analysis. Fifth, the present study analyzed aggregate DOT at the program level and did not differentiate between monotherapy and combination therapy regimens, which is acknowledged as a limitation. However, as the primary stewardship targets—Pip/Taz and meropenem—are typically administered as monotherapy in this institutional context, the majority of DOT reductions observed are likely attributable to monotherapy use. Future patient-level analyses should address this distinction. And the present study was designed as a program-level evaluation of ASP process metrics and antibiotic consumption trends, rather than a patient-level microbiological outcomes study. Individual pathogen data and infection diagnoses were not systematically collected, which is acknowledged as a limitation. However, the appropriateness of carbapenem and Pip/Taz use at this institution is supported by the institutional antibiogram and the prior authorization process, which requires clinical justification at the point of prescription. Additionally, a systematic quantitative analysis of rejection rationale was not feasible due to the small number of rejection cases (monthly mean: 20.1 over 14 months) and the informal nature of physician feedback collection. The absence of systematic resistance surveillance data represents an additional limitation. A recently published interrupted time-series analysis demonstrated that enhanced infection control practices could significantly reduce the incidence of imipenem-resistant *Pseudomonas aeruginosa* [24], suggesting that coupling stewardship interventions with robust resistance monitoring would provide stronger evidence of ecological impact. Similarly, Li et al. demonstrated using ITS analysis that enhanced infection control measures—including weekly active surveillance and contact isolation—were associated with significant reductions in hospital-acquired CROs even amid increasing carbapenem consumption, underscoring the independent contribution of infection control activities to resistance trends [25]. Future iterations of our ASP should incorporate systematic surveillance of carbapenem-resistant *Enterobacterales* and *Pseudomonas* species to determine whether reductions in consumption are associated with measurable declines in institutional resistance rates.

Future studies should expand on the aggregate microbiological findings reported here by incorporating patient-level MDRO acquisition data and clinical outcome measures—including in-hospital mortality, length of stay, and infection-attributable outcomes—to more rigorously quantify the downstream clinical impact of sustained ASP interventions. Future research should also investigate how the reductions in antibiotic consumption demonstrated in this study translate into changes in institutional antimicrobial resistance patterns, as establishing this link remains a critical unmet objective of the current program. Finally, multicenter studies across hospitals of varying sizes and resource levels are warranted to determine whether the EMR-based, small-team model demonstrated here can be replicated and scaled within the Korean healthcare system and beyond.

## 4. Materials and Methods

### 4.1. Study Design and Setting

This retrospective, single-center, longitudinal observational study was conducted from January 2025 to February 2026 at a tertiary university hospital in the Republic of Korea. This study analyzed the sustained performance of the Antimicrobial Stewardship Program (ASP) over a 14-month period by incorporating 10 months of follow-up data into the initial 4-month implementation-phase data reported in a previous study [11]. Throughout the study period, ASP activities were managed by a multidisciplinary team comprising infectious disease specialists, clinical pharmacists, and specialized ASP nurses. The team initially consisted of two infectious disease specialists, one clinical pharmacist, and one ASP nurse; one additional dedicated ASP pharmacist was recruited in January 2026. The ASP was operated without additional capital investment in information technology, as the prior authorization and monitoring functions were implemented within the existing institutional EMR infrastructure. Personnel costs for the ASP team were supported through institutional funding allocated under the national Antimicrobial Resistance Management Program administered by the Korea Disease Control and Prevention Agency (KDCA), which provides dedicated financial support to participating hospitals for stewardship personnel and operational costs.

### 4.2. ASP Interventions

ASP interventions targeted all restricted antibiotic prescriptions across all clinical departments. A system of prior authorization and real-time monitoring through the electronic medical record (EMR) system was implemented for broad-spectrum antibiotics designated by the Pharmacy and Therapeutics Committee, including carbapenems (meropenem, ertapenem, and imipenem/cilastatin), glycopeptides, and oxazolidinones. In addition, prospective audit and feedback (PAF) were performed for both restricted antibiotic prescriptions and cases of prolonged antibiotic use exceeding 14 days. The 14-day threshold for triggering PAF review of prolonged prescriptions was established by the institutional Pharmacy and Therapeutics Committee, based on the premise that antibiotic courses extending beyond two weeks are associated with increased risk of adverse drug events, *Clostridioides difficile* infection, and selection of resistant organisms, and are therefore considered candidates for ASP review regardless of antibiotic class [31,32]. When a prescription was deemed inappropriate, the ASP team communicated recommendations for dose adjustment or discontinuation directly to the prescribing physician via the EMR messaging function.

Antibiotic selection at this institution is guided by annual institutional antibiograms and local susceptibility data updated by the clinical microbiology laboratory. ASP recommendations for de-escalation or discontinuation were made in accordance with these institutional susceptibility profiles, national treatment guidelines, and individual patient clinical data reviewed through the EMR. When a prescription was rejected, the rationale for rejection was communicated to the prescribing physician via EMR messaging or direct contact. In cases where the physician disagreed with the ASP recommendation and maintained the prescription, informal feedback was collected through direct communication; in the majority of such cases, physicians indicated that they considered the antibiotic clinically necessary based on their individual assessment of the patient’s condition. In addition to PAF and prior authorization, the ASP team implemented several complementary stewardship practices consistent with established best practice recommendations throughout the study period [2]. These included the following: (1) periodic educational sessions for each clinical department covering institutional antibiotic guidelines, appropriate antibiotic selection, and optimization strategies including intravenous-to-oral (IV-to-PO) conversion; and (2) regular multidisciplinary committee meetings at which monthly antibiotic consumption metrics and stewardship performance indicators were reviewed and reported to departmental leadership and the hospital infection control committee.

### 4.3. Outcome Measures

The primary outcomes were the monthly rejection rate of restricted antibiotic prescriptions—defined as the proportion of restricted antibiotic requests not approved by the ASP team—and the intervention acceptance rate, defined as the proportion of ASP recommendations (for dose adjustment, de-escalation, or discontinuation) that were subsequently reflected in the actual prescriptions by the treating physician. While the rejection rate measures the ASP team’s gatekeeping function at the point of initial prescription authorization, the acceptance rate measures clinician adherence to post-hoc PAF recommendations and thus captures a distinct and complementary dimension of prescribing behavior change. The secondary outcome was the monthly Days of Therapy (DOT) for meropenem and piperacillin/tazobactam (Pip/Taz), both identified as priority targets for stewardship intervention. Although Pip/Taz was not classified as a restricted antibiotic at this institution, it was continuously monitored throughout the study period as a key optimization target—meaning that the ASP team proactively reviewed Pip/Taz prescriptions during routine PAF rounds and provided recommendations for de-escalation or discontinuation where appropriate, independent of the formal prior authorization process. Meropenem was selected as the representative carbapenem for DOT analysis, consistent with the approach of the prior study, given its predominant use among carbapenem agents at this institution—meropenem accounted for 81.0% of total carbapenem prescription requests throughout the 14-month study period, compared to 5.5% for imipenem/cilastatin and 3.6% for ertapenem. DOT was expressed as the number of antibiotic treatment days per 1000 patient–days to allow standardized comparison across months with varying census volumes. Patient–days were calculated as the sum of all inpatient days across all clinical units, including both ICU and general wards, without differential weighting. ICU and general ward days were treated equally in the denominator, as the primary objective was to monitor institution-wide antibiotic consumption trends rather than to compare unit-specific utilization.

### 4.4. Statistical Analysis

For statistical analysis, data were categorized into two phases: the implementation phase (January–April 2025) and the stabilization phase (May 2025–February 2026). An independent *t*-test was used to compare mean rejection rates, acceptance rates, and DOT values between the two phases. Pearson correlation analysis was performed to assess the relationship between the monthly intervention acceptance rate and antibiotic consumption. All statistical analyses were performed using IBM SPSS Statistics version 26.0 (IBM Corp., Armonk, NY, USA), and a *p*-value of less than 0.05 was considered statistically significant.

### 4.5. Ethical Approval

This study was approved by the Institutional Review Board (IRB) of Inje University Haeundae Paik Hospital (IRB No. 2026-03-028-002). Patient consent was waived due to the retrospective nature of the study and the use of de-identified data, as approved by the IRB.

## 5. Conclusions

This study confirms the extended follow-up sustainability and clinical impact of a multidisciplinary ASP at a tertiary hospital in South Korea over a 14-month observation period, extending the feasibility findings of our prior study into a fully established stabilization phase.

The clinician acceptance rate increased significantly from 72.0% to 81.2% (*p* = 0.035), accompanied by a statistically significant reduction in Pip/Taz DOT (169.4 → 151.8 per 1000 patient–days, *p* = 0.002). The significant negative correlation between acceptance rate and Pip/Taz consumption (r = −0.625, *p* = 0.017) confirms that behavioral change driven by prospective audit and feedback was the primary mechanism of reduction. The downward stabilization of the restricted antibiotic rejection rate (3.8% → 2.6%) despite a 37.3% increase in prescription volume further reflects a durable improvement in institutional prescribing culture. A year-over-year reduction in meropenem DOT of 7.5% (54.8 → 50.7 per 1000 patient–days), alongside the absence of compensatory increases in other antibiotic classes and stable-to-improving MDRO and CDI surveillance metrics, suggests that broad-spectrum antibiotic overuse is being progressively and safely curtailed.

These findings demonstrate that a well-structured EMR-based ASP can achieve extended follow-up sustainability and clinically meaningful reductions in broad-spectrum antibiotic consumption even with a small multidisciplinary team and limited infrastructure—provided it is supported by consistent institutional commitment and a collaborative clinical culture. Sustained investment in ASP operations remains essential for safeguarding the efficacy of last-resort antibiotics and combating the global threat of antimicrobial resistance.

## Figures and Tables

**Table 1:** Trends in Restricted Antibiotic Prescription Requests and Rejection Rates.

Analysis Period	Monthly Total Requests (Average)	Monthly Rejection Cases (Average)	Average Rejection Rate (%) [95% CI]
Implementation Phase (January–April 2025)	546.0	20.8	3.8% [3.1–4.7%]
Stabilization Phase (May 2025–February 2026)	749.4	19.8	2.6% [2.3–3.0%]
**Overall (January 2025–February 2026)**	**691.3**	**20.1**	**2.9% [2.6–3.3%]**

Average Rejection Rate calculated as total rejection cases/total requests per phase (pooled method). 95% confidence intervals calculated using the Wilson score method. The difference in rejection rates between the two phases was statistically significant (χ^2^ = 8.048, *p* = 0.005).

**Table 2:** Monthly Carbapenem Prescription Requests and Rejection Rates by Individual Agent (January 2025–February 2026).

Month	Phase	Meropenem			Ertapenem			Imipenem/Cilastatin			Total Carbapenems		
		Req	Rej	Rate (%)	Req	Rej	Rate (%)	Req	Rej	Rate (%)	Req	Rej	Rate (%)
January 2025	Impl.	207	10	4.8	7	1	14.3	16	0	0.0	230	11	4.8
February 2025	Impl.	162	10	6.2	16	0	0.0	12	1	8.3	190	11	5.8
March 2025	Impl.	194	9	4.6	6	1	16.7	18	0	0.0	218	10	4.6
April 2025	Impl.	222	15	6.8	6	0	0.0	24	1	4.2	252	16	6.3
**Impl. Subtotal**		**785**	**44**	**5.6**	**35**	**2**	**5.7**	**70**	**2**	**2.9**	**890**	**48**	**5.4**
May 2025	Stab.	199	5	2.5	16	1	6.3	20	1	5.0	235	7	3.0
June 2025	Stab.	240	5	2.1	4	0	0.0	13	0	0.0	257	5	1.9
July 2025	Stab.	271	14	5.2	16	0	0.0	20	1	5.0	307	15	4.9
August 2025	Stab.	260	11	4.2	11	0	0.0	7	2	28.6	278	13	4.7
September 2025	Stab.	261	8	3.1	15	0	0.0	11	0	0.0	287	8	2.8
October 2025	Stab.	279	31	11.1	8	1	12.5	23	2	8.7	310	34	11.0
November 2025	Stab.	195	16	8.2	11	1	9.1	18	1	5.6	324	18	5.6
December 2025	Stab.	291	21	7.2	7	0	0.0	12	4	33.3	310	25	8.1
January 2026	Stab.	250	17	6.8	12	0	0.0	10	3	30.0	272	20	7.4
February 2026	Stab.	219	9	4.1	5	0	0.0	10	1	10.0	234	10	4.3
**Stab. Subtotal**		**2214**	**120**	**5.4**	**98**	**3**	**3.1**	**134**	**15**	**11.2**	**2814**	**155**	**5.5**
**Overall Total**		**2999**	**164**	**5.5**	**133**	**5**	**3.8**	**204**	**17**	**8.3**	**3704**	**203**	**5.5**

Req = prescription requests; Rej = rejected (not approved); Rate = rejection rate (%). Impl. = Implementation Phase (January–April 2025); Stab. = Stabilization Phase (May 2025–February 2026).

## Data Availability

The data presented in this study are not publicly available due to institutional privacy policies and ethical restrictions governing patient data. The data may be available from the corresponding author upon reasonable request, subject to approval by the Institutional Review Board of Inje University Haeundae Paik Hospital.

## Supplementary Materials

The following supporting information can be downloaded at https://www.mdpi.com/article/10.3390/antibiotics15060525/s1. Figure S1: Monthly incidence of healthcare-associated pathogens during the study period (per 1000 patient–days, January 2025–February 2026). (A) CRE and CDI; (B) MRAB, MRPA, MRSA, and VRE.
