# Supplementary figures and images for: Sustainability and Impact of an Antimicrobial Stewardship Program on Broad-Spectrum Antibiotic Consumption in South Korea: A 14-Month Extended Follow-Up Study
Source: Antibiotics (Basel). 2026 May 22;15(6):525. doi: 10.3390/antibiotics15060525 (PMC13295571; doi:10.3390/antibiotics15060525)

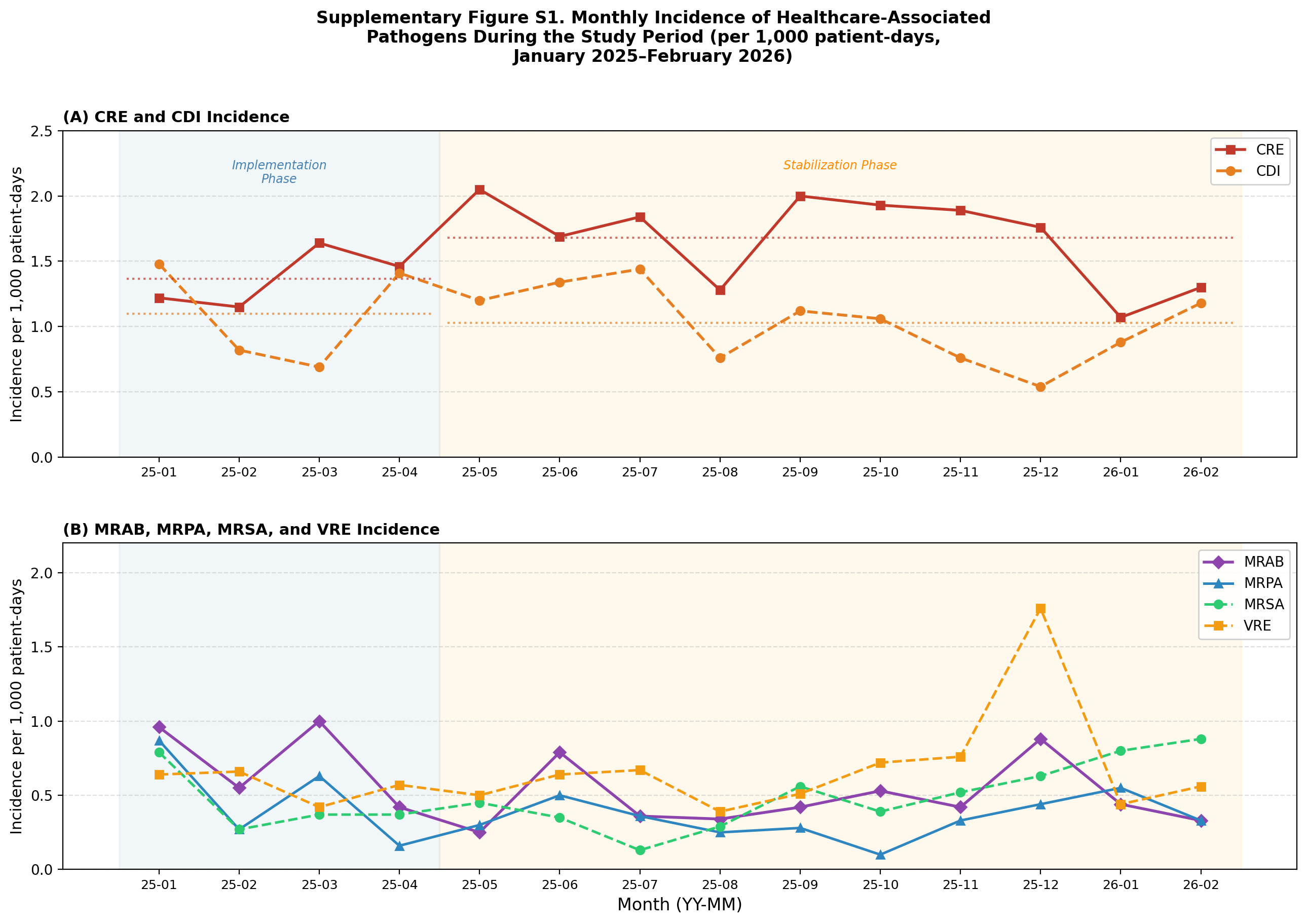

Supplement: Supplementary file 1 [file antibiotics-15-00525-s001.zip › antibiotics-4306853-Supplementary Figure S1.png]
